# Supplementary material for: Experiences of cervical cancer survivors in Chitwan, Nepal: A qualitative study
Source: PLoS One. 2020 Nov 5;15(11):e0234834. doi: 10.1371/journal.pone.0234834 (PMC7644025; doi:10.1371/journal.pone.0234834)
Supplement: Attachment — Submitted filename: Response to reviewers.docx [file pone.0234834.s001.docx]

First of all, we would like to thank the editor and the reviewers for their comments, which have helped significantly to improve the manuscript. We have tried to address all the comments of the reviewers to our possible knowledge.

**Editor evaluation:**

1. Please confirm in the revised manuscript that verbal informed consent was approved by the Institutional Review Committee of BPKMCH, and that this study was conducted in accordance with the Declaration of Helsinki.

Response: We have confirmed it in the revised manuscript that the verbal consent was approved by the review committee, and that this study was conducted in accordance with the Declaration of Helsinki.

2. Abstract: Please shorten your abstract to under 300 words.

Response: We shortened the abstract

3. English correction

Response: Thorough grammar of the manuscript has been improvised and proofed by a native English speaker.

4. Tables: Please move all tables, table legends, and footnotes to the end of the manuscript following the reference list.

Response: Corrected

**Reviewer 1**

1. In introduction section, SARS CoV 2 has been used anonymously to COVID-19; the former term should be reserved for denoting the causative microorganism.

Response: We corrected SARS COV2 as the causative organism.

2. The methodology used - semi-structured interview or questionnaire needs clarification. Validation, pilot testing of the questionnaire, if that's the study tool ?

Response: A semi-structured questionnaire administered by the investigator has been corrected. “The face validation was conducted by two independent experts. The KAP questionnaire was piloted among 15 visitors in the BPKMCH and necessary amendments were done.” Has been added. In statistical analysis “The Cronbach’s alpha coefficient was used to measure the internal consistency of the knowledge questionnaire, which was 0.85 in our sample, indicating excellent reliability.”

3. Title needs to be revised - KAP among cancer patients towards COVID-19.

Response: We have revised the title as “Knowledge, attitudes, and practices of cancer patients towards COVID-19: A cross-sectional study in central Nepal”.

4. Number of questions should be mentioned in tables.

Response: We mentioned the number of questions in the tables.

5. Comparison with the Chinese study on KAP in COVID-19 should be discussed, if that's on cancer patients.

Response: The Chinese study is not on cancer patients, hence comparison with that study was not done.

**Reviewer 2**

1. - The Authors should: - specify what kind of purposive sampling technique was applied to select the participants and why this sampling method was chosen.

Response: We added, “The participants were selected via purposive sampling where we interviewed all available at our convenient time”.

2. - increase data on global mortality rather than limited to Italy as the following recent meta-analysis suggests. (page 4 bibliography 10). Potere N et al. Acute complications and mortality in hospitalized patients with coronavirus disease 2019: a systematic review and meta-analysis. Crit Care. 2020 Jul 2;24(1):389. doi: 10.1186/s13054-020-03022-1.Crit Care. 2020. PMID: 32616077

Response: The above reference does not have the data on mortality due to COVID-19 among cancer. Hence, an Italian study was used to mention the mortality rate among cancer patients due to COVID-19.

Reviewer 3

1. this manuscript needs modification in terms of grammatical and sentencing errors - a couple of examples from the abstract and introduction are mentioned below. Please review the rest of the manuscript and make changes as necessary.

Response: We have corrected all the suggestions. The revised manuscript is rigorously revised for the grammar and was proofed by a native English speaking friend.
